# Fluorescence *In Situ* Hybridization (FISH)-Based Karyotyping Reveals Rapid Evolution of Centromeric and Subtelomeric Repeats in Common Bean (*Phaseolus vulgaris*) and Relatives

**DOI:** 10.1534/g3.115.024984

**Published:** 2016-02-09

**Authors:** Aiko Iwata-Otsubo, Brittany Radke, Seth Findley, Brian Abernathy, C. Eduardo Vallejos, Scott A. Jackson

**Affiliations:** * Center for Applied Genetic Technologies, University of Georgia, Athens, Georgia 30602; † Department of Agronomy, Purdue University, West Lafayette, Indiana 47907; ‡ Division of Plant Sciences, National Center for Soybean Biotechnology, University of Missouri, Columbia, Missouri 65211; § Department of Horticultural Sciences, University of Florida, Gainesville, Florida 32611

**Keywords:** common bean, karyotyping, fluorescence in situ hybridization, satellite repeats, chromosome evolution

## Abstract

Fluorescence *in situ* hybridization (FISH)-based karyotyping is a powerful cytogenetics tool to study chromosome organization, behavior, and chromosome evolution. Here, we developed a FISH-based karyotyping system using a probe mixture comprised of centromeric and subtelomeric satellite repeats, 5S rDNA, and chromosome-specific BAC clones in common bean, which enables one to unambiguously distinguish all 11 chromosome pairs. Furthermore, we applied the karyotyping system to several wild relatives and landraces of common bean from two distinct gene pools, as well as other related *Phaseolus* species, to investigate repeat evolution in the genus *Phaseolus*. Comparison of karyotype maps within common bean indicates that chromosomal distribution of the centromeric and subtelomeric satellite repeats is stable, whereas the copy number of the repeats was variable, indicating rapid amplification/reduction of the repeats in specific genomic regions. In *Phaseolus* species that diverged approximately 2–4 million yr ago, copy numbers of centromeric repeats were largely reduced or diverged, and chromosomal distributions have changed, suggesting rapid evolution of centromeric repeats. We also detected variation in the distribution pattern of subtelomeric repeats in *Phaseolus* species. The FISH-based karyotyping system revealed that satellite repeats are actively and rapidly evolving, forming genomic features unique to individual common bean accessions and *Phaseolus* species.

Common bean (*Phaseolus vulgaris* L., 2*n* = 2*x* = 22) is a very important edible legume, with more than 23 million tons of annual production (Broughton *et al.* 2003). It is a major source of protein in developing countries in Africa, Asia and Latin America. Common bean belongs to the genus *Phaseolus*, which consist of approximately 50 species, classified into eight clades (Delgado-Salinas *et al.* 2006). The genus *Phaseolus* includes other agronomically important crops, such as runner bean (*P. coccineus*), lima bean (*P. lunatus*), tepary bean (*P. acutifolius*), and year bean (*P. dumosus*). All cultivated species except *P. lunatus* are phylogenetically close, belonging to the Vulgaris group (Delgado-Salinas *et al.* 2006). Most *Phaseolus* species posses 2*n* = 2*x* = 22 chromosomes, indicating stability of the chromosome number; there are, however, reports of chromosomal rearrangements between common bean and other *Phaseolus* species (Broughton *et al.* 2003; Singh 2004; Bonifacio *et al.* 2012).

Common bean is a geographically and phenotypically diverse species. Wild common bean is distributed from northern Mexico to northwestern Argentina (Toro Chica *et al.* 1990). Domesticated common bean consists of two major gene pools (Andean and Mesoamerican) that are distinguished by partial reproductive isolating barriers (Shii *et al.* 1980; Gepts and Bliss 1985; Koinange and Gepts 1992), as well as other characteristics, such as morphology, agronomic traits, and seed proteins (Singh *et al.* 1991). The divergence of the gene pools [> 100,000 yr ago (ya)] predates the domestication events within the individual gene pools (8000 ya), a rather unique scenario among crops (Mamidi *et al.* 2013; Schmutz *et al.* 2014). Following independent domestication events, local adaptation has generated diverse landraces.

To date, numerous molecular cytogenetic studies have been undertaken to investigate the chromosomal structure of common bean and other *Phaseolus* species, and has included rDNA distributions, mapping of single and repetitive BAC clones, and development of cytogenetic maps (Moscone *et al.* 1999; Pedrosa-Harand *et al.* 2006, 2009; Fonsêca *et al.* 2010; Altrock *et al.* 2011; Bonifacio *et al.* 2012). The comparison of cytogenetic maps between Andean (G19833) and Mesoamerican (BAT93) landraces revealed the overall stability of karyotypes between the two gene pools (Bonifacio *et al.* 2012). The major cytological difference between the two gene pools is the distribution of the seven 45S rDNA loci, and three 45S rDNA loci in Andean and Mesoamerican varieties, respectively, suggesting Andean-specific amplification of the rDNA loci after the divergence of the two gene pools (Pedrosa-Harand *et al.* 2006).

We previously reported that there are two different centromere-specific satellite repeats, CentPv1 and CentPv2, that have evolved independently within the genome of Andean landrace G19833 (Iwata *et al.* 2013). Unlike most plant species, which have one species-specific centromeric satellite (Jiang *et al.* 2003), fluorescence *in situ* hybridization (FISH) analysis revealed that CentPv1 dominates eight centromeres; whereas CentPv2 mainly dominates the other three centromeres. Furthermore, we found that both CentPv1 and CentPv2 satellite repeats are homogenized within the chromosomes and, in some cases, significant amounts of chromosome-specific variants occupy specific centromeres, as determined by FISH (Iwata *et al.* 2013). However, it is still uncertain how CentPv1 and CentPv2 have selectively dominated subsets of centromeres within the genome of common bean during the evolution of the genus *Phaseolus*, and how quickly they are evolving.

Based on Southern blot analysis, CentPv1 and CentPv2 are highly conserved in wild and cultivated common bean of the two gene pools, but less so in other *Phaseolus* species, including *P. coccineus*, *P. acutifolius*, and *P. dumosus* (Iwata *et al.* 2013). This indicates that contraction, expansion, or divergence of centromeric satellites occurred after common bean and the *Phaseolus* species diverged—a short evolutionary frame. However, Southern blot analysis does not address whether the contraction or expansion involves alteration of centromeric distribution of the repeats.

In addition to centromeric satellite repeats, David *et al.* (2009) reported a subtelomeric satellite repeat, *khipu*, located at cytologically visible knobs at most chromosomal termini of common bean. *Khipu* units in subtelomeres appear to be frequently exchanged between nonhomologous chromosomes, and expansion of *khipu* elements via local duplication has been suggested (Richard *et al.* 2013). This indicates that *khipu* sequences are actively and rapidly evolving by changing their copy numbers and distribution. If this is the case, we hypothesize there is likely variation in copy number and chromosomal distribution of *khipu* among common bean accessions and between species. The *khipu* sequence is extensively conserved among *Phaseolus* species and especially conserved with high copy numbers among species in the Vulgaris group (David *et al.* 2009).

In order to address how satellite repeats, including centromeric and subtelomeric repeats evolve, we used FISH-based karyotyping approaches to explore their distribution in common bean accessions, and other *Phaseolus* species. FISH-based karyotyping using repetitive DNA probes has been developed in maize, soybean, and *Brassica* species (Kato *et al.* 2004; Albert *et al.* 2010: Findley *et al.* 2010; Xiong and Pires 2011), and has been a powerful tool to distinguish individual chromosomes, and to identify variation in chromosome structure and repeat distributions, both between and within species. In this study, we developed a FISH-based karyotype map based on common bean Andean accession G19833 using centromeric satellite repeats CentPv1 and CentPv2, subtelomeric repeat *khipu*, rDNAs, and chromosome-specific BAC clones. Using this FISH-based karyotyping system, we detected variation in satellite repeats in common bean and other *Phaseolus* species, and discuss the evolution of these chromosomal structures.

## Materials and Methods

### Plant materials

All common bean accessions and other *Phaseolus* species used in this study are listed in Table 1. Plants were grown in a greenhouse to obtain root tips for mitotic chromosome preparations.

**Table 1:** List of *Phaseolus* species used in this study

Species	Common name	Gene pool	Accession number	Gene Pool	Type	Source
*P. vulgaris*	Common bean	Andean	G19833	Andean	Landrace	Schmutz *et al.* 2014
*P. vulgaris*	Common bean	Mesoamerican	PI 633451/BAT93	Mesoamerican	Landrace	USDA ARS*^a^*
*P. vulgaris*	Common bean	Andean	G23580	Andean	Wild	CIAT*^b^*
*P. vulgaris*	Common bean	Mesoamerican	PI535416	Mesoamerican	Wild	USDA ARS
*P. coccineus*	Scarlet runner bean		PI 226594		Cultivated	USDA ARS
*P. dumosus*	Year bean		PI 317574		Landrace	USDA ARS
*P. acutifolius*	Tepary bean		PI 319443		Cultivated	USDA ARS

a United States Department Of Agriculture, Agricultural Research Service (http://www.ars-grin.gov/)

b International Center for Tropical Agriculture (http://ciat.cgiar.org/)

### Cloning of 5S rRNA genes (rDNA)

The 5S rDNA was amplified from genomic DNA using PCR with the following primers: 5′- GGTGCGATCATACCAGCACT-3′ and 5′-AAGTGCAACACGAGGACTTC-3′. A dimer form of 5S rRNA gene was gel-purified using a kit (Qiagen), ligated to a TOPO TA cloning vector, and transformed into the *Escherichia coli* strain top10 (Invitrogen). The insert was sequenced using Big Dye Terminator chemistry (Applied Biosystems) and run on an ABI3730 sequencer.

### Selection of chromosome-specific single- or low-copy BAC clones

Single- or low-copy BAC clones were selected from the G19833 PVGBa BAC library (Schlueter *et al.* 2008). Bng markers (GenBank KM061153–KM061375), located near the end of linkage groups, were used to select corresponding BAC clones using Southern blotting (Table 2; Vallejos *et al.* 1992 unpublished data; Bhakta *et al.* 2015). For chromosomes without Bng markers near the ends of the linkage groups, or for which we could not find single- or low-copy BACs using Bng markers, we performed *in silico* analysis to select chromosome-specific BAC clones. BAC end sequences were used to pull out entire BAC sequences from 11 common bean pseudo-chromosomes (http://www.phytozome.org) using BLASTN with the following criteria: > 99% identity and > 90% query coverage, the BAC end pairs oriented in opposite directions, and putative pairs separated by 50–200 kb (slightly modified from Findley *et al.* 2010). The repetitive portion of BAC sequences were masked using RepeatMasker (http://www.repeatmasker.org/) with low complexity repeats, simple repeats, identified tandem repeats (rDNAs, CentPv1, CentPv2, and *khipu*), and the common bean transposon database (http://www.phytozome.org). We selected BAC clones for FISH probes that had a relatively low repeat content (< 20%), no tandem repeats, and that were located near the ends of pseudo-chromosomes (Table 2).

**Table 2:** BAC clones used for chromosome identification of common bean

Chromosome	Linkage Group	PV_GBa BAC clone	Bng markers	First Base	Last Base	Insert Length (bp)	Physical Position in Pseudo-Chromosomes (%)*^a^*	Total Repeat Content (%)*^b^*
1	H	0043E17	Bng 083	51,533,991	51,677,621	143,631	98.85	15.47
2	D	0073I08	—	42,399,450	42,539,858	140,409	86.6	10.05
3	C	0084M03	—	50,068,422	50,162,332	93,911	95.85	15.14
4	B	0095D15	—	44,627,451	44,745,580	118,130	97.23	14.38
5	E	0101A23	Bng 162	40,054,960	40,179,280	124,321	98.28	15.26
6	G	0043E14	—	29,436,487	29,562,452	125,966	92.25	9.48
7	A	0100C06	Bng 042	3,452,024	3,577,941	125,918	6.79	12.48
8	F	0102L19	Bng 139	172,969	320,543	147,575	0.41	11.41
9	K	0100J23	—	21,306,679	21,417,904	111,226	57.01	16.62
10	I	0103D09	—	41,338,907	41,446,050	107,144	95.65	12.89
11	J	0033F21	Bng 025	6,754,339	6,864,292	109,954	13.33	19.42

a Physical position in pseudo-chromosomes is calculated from the following formula. The median value of the first and last bases/total length of the pseudo-chromosome.

b Total repeat content contains transposon, low complexity repeats, and simple repeats.

### Searching for potential chromosome-specific variants

Multiple alignments of CentPv1 monomers from eight pseudo-chromosomes corresponding to chromosomes 1, 2, 3, 4, 7, 8, 9, and 10 were used to find chromosome-specific variants (Iwata *et al.* 2013).

### Oligonucleotide FISH probes used in this study

The following fluorochrome-labeled oligonucleotides were used as FISH probes (Integrated DNA Technologies). CentPv1: CACATGAAATTGTTTTTCAAAGATA labeled with cyanine 5 (Cy5), CentPv2: CAATAAATTCATGCAACTACCACAA labeled with TEX615, CentPv1_A: GGTTTTTCAAGGGTGTATCATAGGT labeled with FAM (fluorescein), CentPv2_A: CCAATGTCTATCACTACTCTTTGACA labeled with FAM, CentPv1_B: TCAAAGGTATTATCACAAGTGTTCGA labeled with FAM and TEX615, CentPv1_C: TTCATTCATAAGTGTTTCAATCAATT labeled with TEX615, CentPv1_D: ATCTATCATAAGTGTTTCAATCAGTT labeled with TEX615, *khipu*: GACACAGTGACGAATGTCTGGTAAA labeled with TEX615, 5S rDNA: GCACTAATGCACCGGATCCCATCA labeled with TEX615, and 18S rDNA: GTAATTCCAGCTCCAATAGCGTATA labeled with FAM.

### Direct labeling of BAC clones, khipu and rRNA genes

BAC DNA was extracted using a standard Alkali lysis method (Sambrook and Russell 2001). 5S and 18S rDNA (developed and provided by D.A. Johnson at University of Ottawa) was extracted using a QIAprep Spin Miniprep Kit (Qiagen), and amplified by PCR using universal primers to obtain sufficient quantities of the genes. PCR amplicons of CentPv1 and CentPv2 (Iwata *et al.* 2013), rDNA, and BAC DNA were labeled, either with biotin-dUTP or digoxigenin-dUTP, using Biotin- or DIG-Nick Translation Mixes (Roche), respectively. BAC DNA, and amplified 5S rDNA, were labelled directly by nick translation with fluorescein-12-dUTP (Invitrogen) according to Findley *et al.* (2010) and Kato *et al.* (2006). *Khipu* sequence was amplified from genomic DNA of G19833 with a set of primers (5′TTCCACGTAAGAATCTCCAC-3′/5′-AACCAAGGCTATCCTCTACC-3′), and labeled with Texas Red-12-dUTP (Invitrogen).

### Fluorescence *in situ* hybridization

Mitotic chromosome preparations and FISH using oligonucleotide probes were conducted as described in Gill *et al.* (2009) and Findley *et al.* (2010), with the following modifications. Root tips from potted plants were treated with pressurized nitrous oxide for 90 min, fixed in Carnoy’s solution composed of 3:1 ethanol and glacial acetic acid for a day at room temperature, and then stored at 4°. After rinsing the fixed root tips in distilled water, the root tips were digested with enzyme solution containing 1% Pectolyase (MP Biomedicals, LLC), and 2% Cellulase (MP Biomedicals, LLC) in citric buffer (10 mM sodium citrate, 10 mM sodium EDTA, pH 5.5) for 80 min at 37°. The following procedure is the same as reported in Gill *et al.* (2009) and Findley *et al.* (2010). FISH using biotin- or digoxigenin-labeled probes were performed as described in Walling *et al.* 2005. When slides were reprobed with chromosome-specific BAC clones, the slides used for oligonucleotide FISH were soaked in 1x PBS to remove cover slips, and washed three times in 1x PBS for 5 min. each, followed by FISH protocol using biotin- or digoxigenin-labeled probes.

### Microscopy and image processing

Images were collected with a Zeiss Axio Imager M2 microscope, equipped with AxioCam MRm, controlled by Axio Vision 40 V4.8.2.0. Adobe Photoshop CS5 (Adobe Systems Incorporated) was used to produce publication images as described in Findley *et al.* (2010) with slight modification. Aligned chromosome images were produced with the “lasso” tool to trace individual chromosomes, and then orient and align them with the “copy and paste” and “rotation” tools. At least three images were analyzed to confirm the intensity and chromosomal distributions of individual FISH signals.

### Data availability

The authors state that all data necessary for confirming the conclusions presented in the article are represented fully within the article.

## Results

### FISH probes targeted to repeats for use in karyotyping

Using common bean centromeric satellite repeats and their variants (Iwata *et al.* 2013), we first examined the utility of these repeats as FISH probes for a karyotype map using the sequenced Andean accession, G19833 (Schmutz *et al.* 2014; http://www.phytozome.org). For use as karyotyping probes, we designed 25-bp oligonucleotide probes labeled with fluorophores targeted to centromeric repeats CentPv1 and CentPv2, and their variants CentPv1_A and CentPv2_A. CentPv1_A and CentPv2_A are chromosome-specific variants of CentPv1 and CentPv2 (Iwata *et al.* 2013). Using these four oligonucleotides with different fluorophore labels (Cy5-CentPv1, TEX615-CentPv2, FAM-CentPv1_A, and FAM-CentPv2_A), we performed FISH to determine how many chromosomes have unambiguous and distinguishable patterns of hybridization signals (Supplemental Material, Figure S1). We pseudo-colored the merged images of DAPI, FAM, TEX615, and Cy5 in transparent gray, green, red, and blue, respectively. In this way, we were able to distinguish four of the 11 chromosome pairs (later identified as chromosomes 5, 6, 8 and 11).

Next, we designed a 25-bp oligonucleotide probe targeted to *khipu* from the consensus sequences of 92 *khipu* sequences. We tested the *khipu* oligonucleotide probe in accession BAT93 (Mesoamerican type) to examine the consistency of hybridization signals with a previous report (Figure S2; David *et al.* 2009; Richard *et al.* 2013). The *khipu* oligonucleotide probe had minor signals at most ends of the chromosomes, with one pair of strong signals (arrows in Figure S2B), and a secondary pair of signals (arrowheads in Figure S2B). Later, we confirmed that the strongest signals are on the short arm of chromosome 4, and the secondary signals on the long arm of chromosome 11, in agreement with David *et al.* (2009) and Richard *et al.* (2013).

rDNA is known to be present at high copy number in plant genomes, and is often used as FISH markers. We used oligonucleotide FISH probes targeted to 18S rDNA and 5S rDNA, but found only faint or no signals from these probes for unknown reasons (data not shown). Thus, we decided to use probes labeled by directly the nick translation method to detect rDNA loci for further experiments.

**Figure 1:**
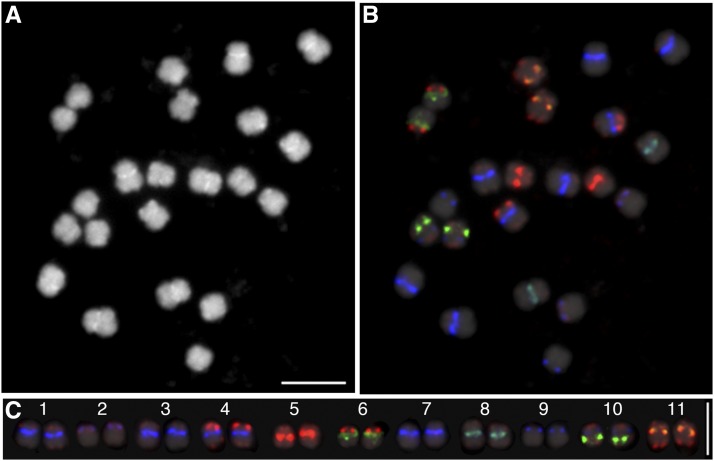
A karyotype map on G19833 metaphase chromosomes. (A) Chromosomes counter-stained with DAPI. (B) FISH image with a probe mixture of Cy5-CentPv1 (blue), FAM-CentPv1_A (green), TEX615-CentPv2 (red), FAM-CentPv2_A (green), TEX615-*khipu* (red), and fluorescein-5S rDNA (green). (C) Homologous chromosome pairs aligned according to chromosome numbers based on reprobing with chromosome-specific BAC clones. Bars represent 5 µm.

We next performed FISH with a mixture of oligonucleotide probes (Cy5-CentPv1, TEX615-CentPv2, FAM-CentPv1_A, FAM-CentPv2_A, and TEX615-*khipu*), and nick-translated 5S rDNA probe labeled with fluorescein. We did not add the 18S rDNA probe in the mixture because it showed seven strong hybridization signals in G19833 overlapping with several *khipu*, CentPv1, and CentPv2 signals, which prevented us from analyzing the distribution of the satellite repeats (data not shown). Using a mixture of the six probes above, all 11 chromosomes of G19833 could be distinguished with different signal locations and intensities (Figure 1, A and B).

### Identification of individual chromosomes of common bean

In order to identify individual chromosomes labeled with four CentPv repeats, *khipu*, and 5S rDNA, we reprobed the slides with chromosome-specific BAC clones from the G19833 PV_GBa BAC library (Schlueter *et al.* 2008). We identified chromosome-specific BACs either experimentally or by *in silico* analysis. Southern blotting was conducted using Bng clones anchored near the ends of individual linkage groups (Vallejos *et al.* 1992; Bhakta *et al.* 2015; unpublished data). Based on *in silico* analysis, 12,151 out of 48,000 PV_GBa BAC clones were anchored to 11 pseudo-chromosomes, and their entire BAC sequences were obtained. Because repetitive sequences in BAC clones can complicate chromosome-specific FISH hybridization, we analyzed the repeat content of these BAC clones, and selected those with relatively low repeat content. Table 2 summarizes the selected BAC clones for individual chromosomes that showed unambiguous FISH signals on their chromosomes.

Chromosome-specific BAC-FISH allowed us to identify all 11 chromosomes as correlated with FISH signal patterns of four CentPv components, *khipu*, and 5S rDNA. Images of individual homologous chromosome pairs were aligned according to their chromosome numbers (Figure 1C). This revealed that CentPv1 is present at centromeres 1, 2, 3, 4, 7, 8, 9, and 10, with its variant, CentPv1_A present only at centromere 8. The four strongest CentPv1 FISH signals were observed at centromeres 1, 3, 4, and 7, suggesting a higher copy number of CentPv1 in these centromeres. CentPv2 is present at centromeres 5, 6, and 11, with its variant CentPv2_A at centromere 11. Occasionally, weak CentPv2_A signals were also detected on the centromere of chromosome 5. Although these FISH signals and intensities were sufficient to distinguish all 11 chromosomes, three chromosomes pairs (chromosome 1, 3, and 7) had similar signal patterns that occasionally made them difficult to distinguish. Therefore, we added more probes to unambiguously distinguish these three chromosome pairs. In addition, the oligo-based probe of *khipu* was not always detectable, particularly at chromosomal termini with low copy numbers; therefore, we moved from an oligo-based to a PCR-amplified *khipu* probe for further experiments.

### FISH-based karyotype map established for common bean

**Figure 2:**
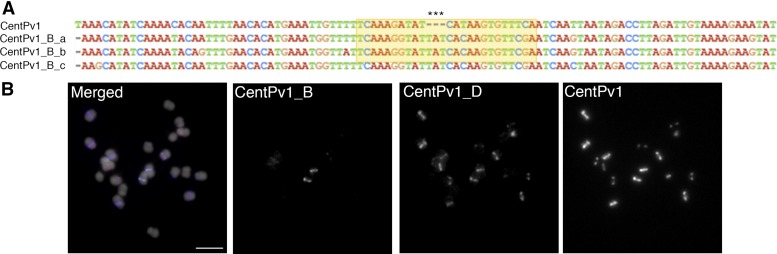
FISH analysis of CentPv1 variant, CentPv1_B, and CentPv1_D. (A) Multiple alignments of CentPv1 and its variant, CentPv1_B. The 25-bp oligonucleotide CentPv1_B for FISH was designed from the region in the yellow rectangle targeted to polymorphisms shown by asterisks. (B) FISH image with CentPv1_B (green), CentPv1_D (red), and CentPv1 (blue). CentPv1_B signals localized at one pair of centromeres. Cent Pv2_D signals overlapped with all CentPv1 signals. Bar represents 5 µm.

**Figure 3:**
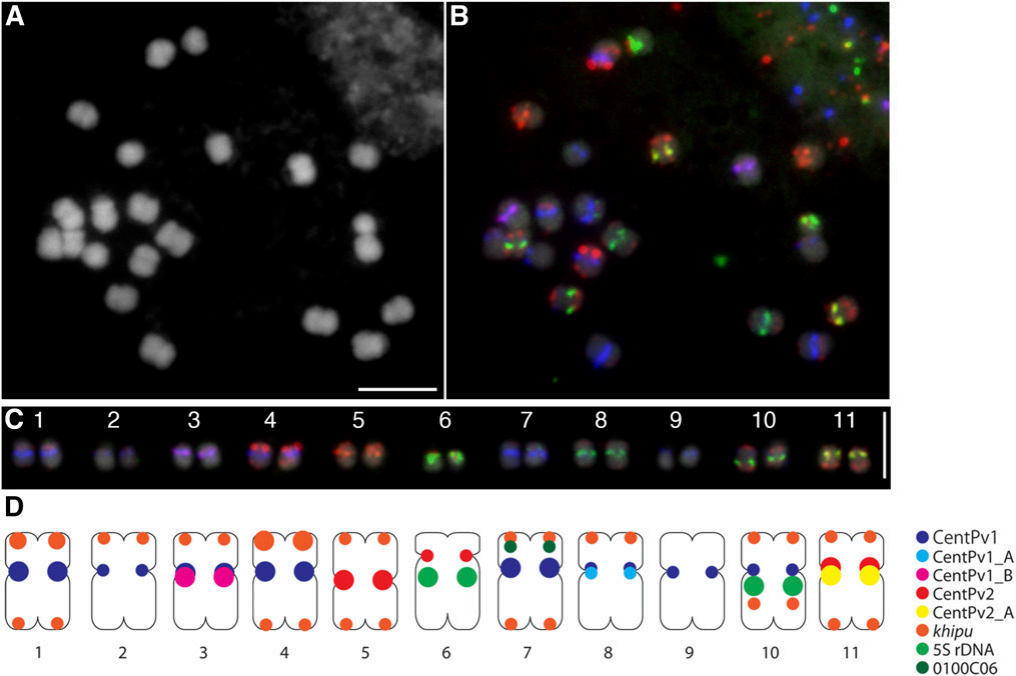
A final version of karyotype map on G19833 metaphase chromosomes. (A) Chromosomes counter-stained with DAPI. (B) FISH image with a probe mixture of Cy5-CentPv1 (blue), FAM-CentPv1_A (green), TEX615-CentPv1_B (red), TEX615-CentPv2 (red), FAM-CentPv2_A (green), *khipu* labeled with TexRed (red), 5S rDNA labeled with fluorescein (green), and 0100C06 labeled with fluorescein (green). (C) Homologous chromosome pairs aligned according by chromosome numbers. Scale bars represent 5 μm. (D) Illustration of a karyotype map for G19833. The size of circle reflects the intensity of each signal.

In order to identify potential chromosome-specific variants of CentPv1 that could help distinguish chromosome 1, 3, and 7, we compared multiple-aligned CentPv1 monomer sequences from eight pseudo-chromosomes corresponding to chromosome 1, 2, 3, 4, 7, 8, 9, and 10. We found three different repeat variants from three pseudo-chromosomes: 1, 3, and 7; 25-bp oligonucleotides targeted to polymorphic regions of each repeat variant were used as a FISH probe to examine their distribution. Only the oligonucleotide targeted to a repeat variant found in pseudo-chromosome 3 hybridized specifically to chromosome 3 (named as CentPv1_B; Figure 2), thereby helping us to distinguish chromosome 3 from chromosomes 1 and 7. The other two oligonucleotides with complementary sequences found in pseudo-chromosome 1 and 7 (named as CentPv1_C and CentPv1_D, respectively; Figure S3) hybridized to multiple chromosomes with different intensities (CentPv1_D in Figure 2, and CentPv1_C in Figure S3). Because we could not find a CentPv1 variant that helped distinguish chromosome 1 from 7, we instead added a chromosome 7-specific BAC clone, 0100C06, directly labeled with fluorescein, which hybridized to the short arm of chromosome 7. This probe set established the final version of a karyotype cocktail for common bean. This set comprised three probes from CentPv1 (Cy5-CentPv1, FAM-CentPv1_A, and TEX615-CentPv1_B), two from CentPv2 (TEX615-CentPv2 and FAM-CentPv2_A), 5S rDNA labeled with fluorescein, *khipu* labeled with Texas Red, and a chromosome7-specific BAC clone, 0100C06 labeled with fluorescein (Figure 3).

### Comparison of FISH-based karyotype maps within common bean

Repetitive DNA sequences are known to be fast-evolving (reviewed in Mehrotra and Goyal 2014). In maize, there is distinct variation of repeats in their copy number and presence among maize lines that diverged within the past 6000–10,000 yr (Kato *et al.* 2004; Albert *et al.* 2010). Because the developed karyotype map of common bean uses centromeric and subtelomeric satellite repeats, this karyotyping strategy has the potential to investigate satellite repeat evolution not only within common bean, but also in allied species where satellite repeats are conserved.

**Figure 4:**
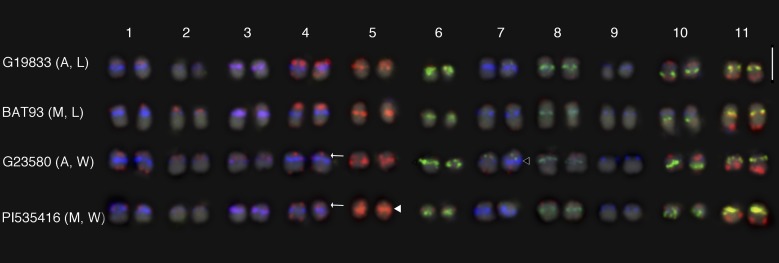
FISH based karyotype maps of G19833, BAT93, G23580 and PI535416. All homologous chromosome pairs were distinguished using a probe mixture of Cy5-CentPv1 (blue), FAM-CentPv1_A (green), TEX615-CentPv1_B (red), TEX615-CentPv2 (red), FAM-CentPv2_A (green), *khipu* labeled with Texas Red (red), 5S rDNA labeled with fluorescein (green), and chromosome7 specific BAC clone, 0100C06 labeled with fluorescein (green). A, Andean gene pool; M, Mesoamerican gene pool; L, Landrace; W, Wild. Scale bar represents 5 µm. Arrows indicate weaker *khipu* signals on chromosome 4S of wild common bean. Closed arrowhead shows strong signals of CentPv2 on chromosome 5, indicating increased copy number of the CentPv2. Open arrowhead shows copy number variation of CentPv1 between homologous chromosome of chromosome 7.

We applied the same probe mixture to Mesoamerican accession, BAT93, and compared it with the karyotype map of Andean accession, G19833. With the same probe mixture, all 11 chromosomes were distinguished in BAT93. A detailed comparison between BAT93 and G19833 was made using aligned chromosome images (Figure 4). The overall signal patterns of centromeric and subtelomeric satellite repeats in BAT93 were similar to those of G19833. Five components of centromeric satellite repeats showed the same signal patterns between G19833 and BAT93, suggesting chromosomal distributions of these repeats were fixed before the divergence of Andean and Mesoamerican gene pools, ∼100,000 ya.

The karyotyping probe mixture was also hybridized to wild common bean from the Andean (G23580) and Mesoamerican (PI535416) gene pools (Figure 4). Overall, the repeat distributions were conserved, and it was possible to clearly distinguish all of the chromosomes, except chromosomes 1 and 4. In G19833 and BAT93, chromosomes 1 and 4 were distinguished by the intensity of *khipu* signals; *khipu* signals at the short arm of chromosome 4 are much stronger than those of chromosome 1. In wild accessions, *khipu* signals at the long arm of chromosome 11 were the strongest, whereas the signal at the short arm of chromosome 4 was relatively weaker. This variation indicates that *khipu* is evolving actively within the common bean. We conducted a subsequent FISH on these same slides using chromosomes 1- and 4- specific BAC clones to confirm their identity, and aligned the chromosomes (Figure S4).

While we detected consistency of chromosomal distribution of five components of centromeric satellite repeats in these wild accessions, we did detect copy number variation of CentPv1 and CentPv2. We consistently detected different signal intensities of CentPv1 at chromosome 7 between homologous chromosomes of G23580, indicating heterozygosity of the repeat distribution (open arrowhead in Figure 4). Because in other accessions, chromosome 7 has high copy numbers of CentPv1, it seems that the copy number of CentPv1 of one of the homologous chromosomes 7 is reduced in G23580. In PI535416, we consistently detected stronger signals of CentPv2 at chromosome 5 in comparison to chromosome 5 of other accessions, indicating an increase in copy number specific to the PI535416 (closed arrowhead in Figure 4).

High variation of 18S rDNA loci within common bean was previously reported (Pedrosa-Harand *et al.* 2006). For example, G19833 has seven loci (chromosomes 1S, 3S, 4L, 5L, 6S, 9S, and 10L), while BAT93 has only three loci (chromosomes 6S, 9S, and 10L) of 18S rDNA (Fonseca *et al.* 2010; Altrock *et al.* 2011). Here, we determined the positions of 18S rDNA loci in wild accessions by performing the second FISH using 18S rDNA as a probe after karyotyping (Figure S4). There are seven loci in G23580, and three loci in PI535416, supporting extensive amplification of 18S rDNA in the Andean lineage (Pedrosa-Harand *et al.* 2006). In Mesoamerican accessions, the chromosomal distributions of 18S rDNA loci were consistent between BAT93 and PI535416. In Andean accessions, while the number of loci was the same between G19833 and G23580, the locations varied, as the 18S rDNA locus on chromosome 4L was instead found on chromosome 11L (Figure S4).

### Conservation and variation of the repeats in related Phaseolus species

**Figure 5:**
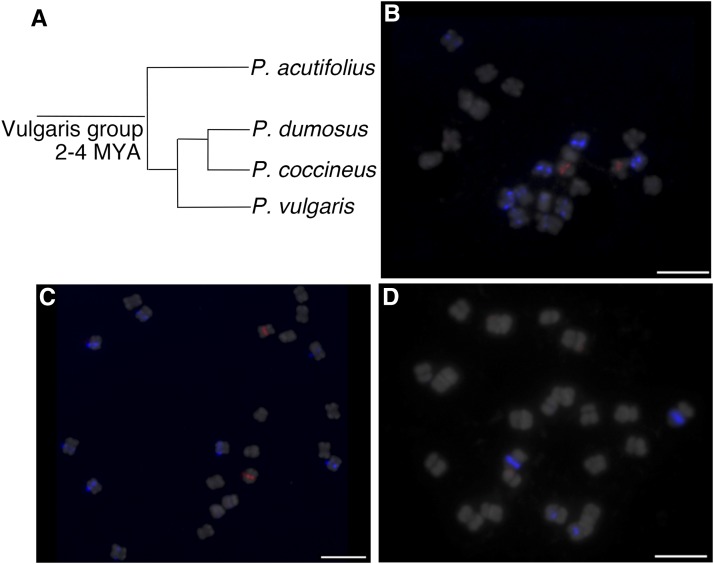
(A) Phylogenetic relationship among common bean (*Phaseolus vulgaris*), *P. coccineus*, *P. dumosus*, and *P. acutifolius*. The tree is simplified from Delgado-Salinas *et al.* (2006). FISH analysis of Cy5-CentPv1 (blue) and TEX615-CentPv2 (red) on *P. coccineus* (B), *P. dumosus* (C) *P. acutifolius* (D). Scale bar represents 5 µm.

We previously confirmed by Southern blot analysis that CentPv1 and CentPv2 are conserved in the Vulgaris group that diverged 2–4 million ya (Iwata *et al.* 2013). Weak hybridization signals of CentPv1 and CentPv2 on Southern blots indicated dramatic reductions, or divergence in copy numbers of these repeats, in the other *Phaseolus* species as compared to common bean. The next question was whether the chromosomal distributions of these repeats were conserved in other *Phaseolus* species. We tested the centromeric repeats, CentPv1 and CentPv2, on *P. dumosus*, *P. coccineus*, and *P. acutifolius* (Figure 5A). Interestingly, FISH using Cy5-CentPv1 and TEX615-CentPv2 showed variation in their chromosomal distribution, depending on the species: CentPv1 repeats were present only at five centromeres, with strong signals at two centromeres, middle-strength signals at two centromeres, and weak signals at one centromere in *P. coccineus* and *P. dumosus*. CentPv2 was localized to only one centromere, with strong signals in *P. coccineus* and *P. dumosus* (Figure 5, B and C). There were CentPv1 signals on two centromeres, and no CentPv2 signals in *P. acutifolius* (Figure 5D). We also tested probes of CentPv variants, FAM-CentPv1_A, FAM-CentPv2_A, and FAM-CentPv2_B, but detected no signals from these probes, indicating the absence of these variants in these three *Phaseolus* species. Because oligo-based probes target only 25-bp, and we wanted to test whether the entire sequences of CentPv1 and CentPv2 were conserved, we also conducted FISH using PCR products covering the entire region of CentPv1 and CentPv2. There were CentPv1 signals on five centromeres in *P. coccineus* and *P. dumosus*, consistent with oligo-based FISH results (Figure S5). However, we could not find CentPv1 signals in *P. acutifolius*, and detected only very weak or no signals of CentPv2 in cells of all three species. This indicates that the entire sequences of CentPv1 and CentPv2 are either not conserved, or are present at very low copy number, in *P. acutifolius*, and the same for CentPv2 in all three species, and the 25-bp sequences of oligonucleotide probes appear to represent a more conserved sequence of the repeat (data not shown).

**Figure 6:**
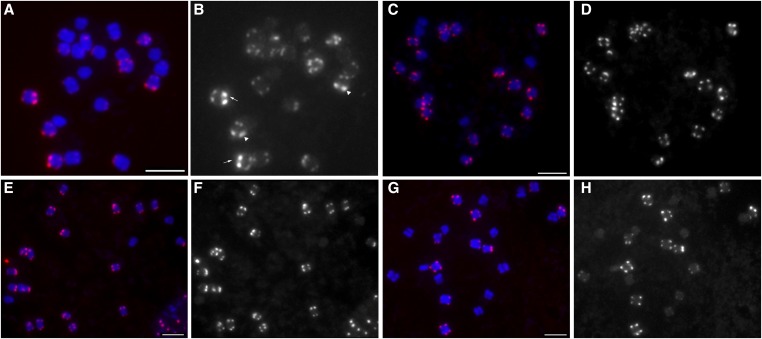
FISH analysis of *khipu* on G19833 (A and B), *P. coccineus* (C and D), *P. dumosus* (E and F) and P. *acutifolius* (G and H). Mitotic metaphase chromosomes were counter-stained with DAPI (blue) merged with *khipu* signals. Scale bars indicate 5 µm. Panel (A) shows the strongest signals of *khipu* on chromosome 4S. Arrowheads show the second strongest signals of *khipu* on chromosome 11L.

We also examined *khipu* distribution in the other *Phaseolus* species. Based on Southern analysis, *khipu* was conserved across the *Phaseolus* species with varying copy numbers. *Khipu* was conserved at high copy numbers in *P. dumosus* and *P. parvifolius* in the Vulgaris group (David *et al.* 2009), although the chromosomal distribution remained unknown. Based on FISH analysis using PCR-amplified *khipu* as a probe, we confirmed that *khipu* is present at subtelomeric regions of most of the chromosomes with different signal intensities in *P. coccineus*, *P. dumosus*, and *P. acutifolius* (Figure 6). We did detect variation in *khipu* distributions as compared to common bean. Based on our observations, *khipu* signals in *P. coccineus* and *P. dumosus* were distributed more uniformly at most chromosomal termini, and we did not detect any distinctively strong signals such as on chromosomes 4S (arrows in Figure 6A) and 11L (arrowheads in Figure 6A) in common bean. In *P. acutifolius*, five pairs of chromosomes have very weak or no *khipu* signals. Together with the FISH analysis of *khipu* on wild common bean, we concluded that *khipu* sequences evolve via changes in copy number and distribution over relatively short evolutionary time frames.

## Discussion

The establishment of a karyotype map is essential for chromosome studies. It requires high quality chromosome spreads (*e.g.*, no overlap of chromosomes, good chromosome morphology, and little or no cytoplasm), and reliable FISH markers, especially for identification of small chromosomes. In this study, we developed a FISH-based karyotype map of common bean using a set of repetitive sequences: CentPv1, CentPv1_A, CentPv1_B, CentPv2, CentPv2_A, *khipu*, 5S rDNA, and chromosome-specific BAC clones hybridized to mitotic metaphase chromosomes. There are several advantages to our FISH-based karyotype map. First, probes of centromere satellite repeats, CentPv1, and CentPv2 enable one to position centromeres easily and accurately in chromosomes. This study enabled us to characterize the distribution and relative copy number of CentPv1 and CentPv2 in individual chromosomes of the common bean. Second, because we used conserved satellite repeats found at centromeres and subtelomeres of the Vulgaris group, we were able to apply this karyotyping system to wild and domesticated Mesoamerican and Andean common bean, as well as to other closely related *Phaseolus* species. Third, the karyotype map has been integrated with the genetic map using chromosome-specific, genetically anchored, BACs.

Common bean is a geographically and phenotypically diverse species, consisting of two major gene pools: Andean and Mesoamerican. Wild accessions of the Mesoamerican gene pool were found to have higher genetic diversity than their Andean counterparts, evidence of a tighter bottleneck in the Andean gene pool (Koenig and Gepts 1989; Koenig *et al.* 1990; Velasquez and Gepts 1994; Chacon *et al.* 2007; Bitocchi *et al.* 2012). One of our interests in this study was to determine the extent of chromosomal variation for an informative set of repetitive DNA sequences, as a corollary to genetic variation within and between the gene pools. Polymorphisms in 45S rDNA loci among plant species are frequently seen (Chung *et al.* 2008; Hamon *et al.* 2009; Robledo and Seijo 2009), even within species (Chung *et al.* 2008; de Moraes *et al.* 2007; Hayasaki *et al.* 2001; Raskina *et al.* 2004; Pedrosa-Harand *et al.* 2006). Pedrosa-Harand *et al.* (2006) showed the extensive variation of 45S rDNA loci within common bean, *e.g.*, the number of rDNA loci was amplified in Andean lineage. Fewer studies have analyzed satellite repeat distributions within and among species. In maize, a very genetically diverse species, variation in copy number and/or positions in knob-related repeats, and the centromeric satellite repeat, CentC, were observed within maize, and among closely related species, using FISH-based karyotype maps (Kato *et al.* 2004; Albert *et al.* 2010). Unlike maize, common bean is a mostly autogamous selfing species, but geographically dispersed with two distinct gene pools. We were interested in determining how the geographical distribution may have affected chromosomal evolution, especially of rapidly evolving repeats, within the genus and species. The comparison of FISH-based karyotypes showed that the overall repeat distribution and chromosome structure were very similar between Andean and Mesoamerica landraces, a pattern that is indicative of the fixation of the repeats before divergence of the two gene pools ∼100,000 ya.

The majority of eukaryotes have centromeric satellite repeats; however, the function, emergence, and evolution of centromeric satellite repeats are still ambiguous. Most neocentromeres (ectopic centromeres formed at previously noncentromeric chromosomal regions) form in nonrepetitive chromosomal regions, indicating that satellite repeats are not necessary to form centromeres *de novo* (Marshall *et al.* 2008). In a few cases, such as chicken and potato, different centromere structures (repeat-based *vs.* repeat-free) coexist within the same genome (Shang *et al.* 2010; Gong *et al.* 2012), which may represent a transition stage of repeat-free to repeat-based centromeres. Although all common bean centromeres have satellite repeats, we hypothesize that the two centromeric repeats might not yet be fixed in the genome, and ultimately they will reach a “stable” state where one centromeric repeat will dominate all 11 centromeres. In fact, we observed copy number variation for CentPv1 and CentPv2 in wild common bean accessions, which might indicate that these repeats are still actively and rapidly evolving by increasing/decreasing their copy numbers at specific centromeric locations. In addition, the presence of variants of CentPv1 and CentPv2 limited to common bean indicates the rapid evolution of the repeats after its divergence from other *Phaseolus* species. The presence of CentPv1 and CentPv2 in a fewer number of chromosomes in other *Phaseolus* species than in *P. vulgaris* may also favor the hypothesis that centromeres evolve from repeat-free to repeat-based structures. From this study, it is not clear that the centromeres devoid of CentPv1 and CentPv2 signals are repeat-free, or have been replaced with other satellite repeats. In either case, subsets of centromeres are actively evolving in the genus *Phaseolus*, while the others are conserved within the genus *Phaseolus*. Whether this has occurred by selective pressure or by random genetic drift remains to be determined.

*Khipu* is a subtelomeric repeat originally identified from disease resistance gene (*R*) clusters (David *et al.* 2009). The *B4* and *Co-2 R* resistance loci at the end of LG-B4 and LG-B11 contain a Coiled-Coil-Nucleotide-Binding-Site-Leucine-Rich-Repeat (CNL) gene, and *Khipu* is interspersed between CNL sequences in *R* gene clusters, and present at adjacent heterochromatic knobs. Interestingly, *khipu* localization is not only restricted to these two regions, but is also found at most chromosome termini, indicating possible ectopic recombination in subtelomeric regions between nonhomologous chromosomes (David *et al.* 2009). In this study, we found that the distribution of *khipu* varies within common bean, and among *Phaseolus* species, in the Vulgaris group. The strongest *khipu* signals at chromosome 4S were restricted to common bean landraces, regardless of gene pools. Differences in *khipu* distribution among species further supports frequent ectopic recombination between nonhomologous chromosomes in subtelomeric regions during the evolution of *Phaseous* species, as proposed by David *et al.* (2009).

**Figure 7:**
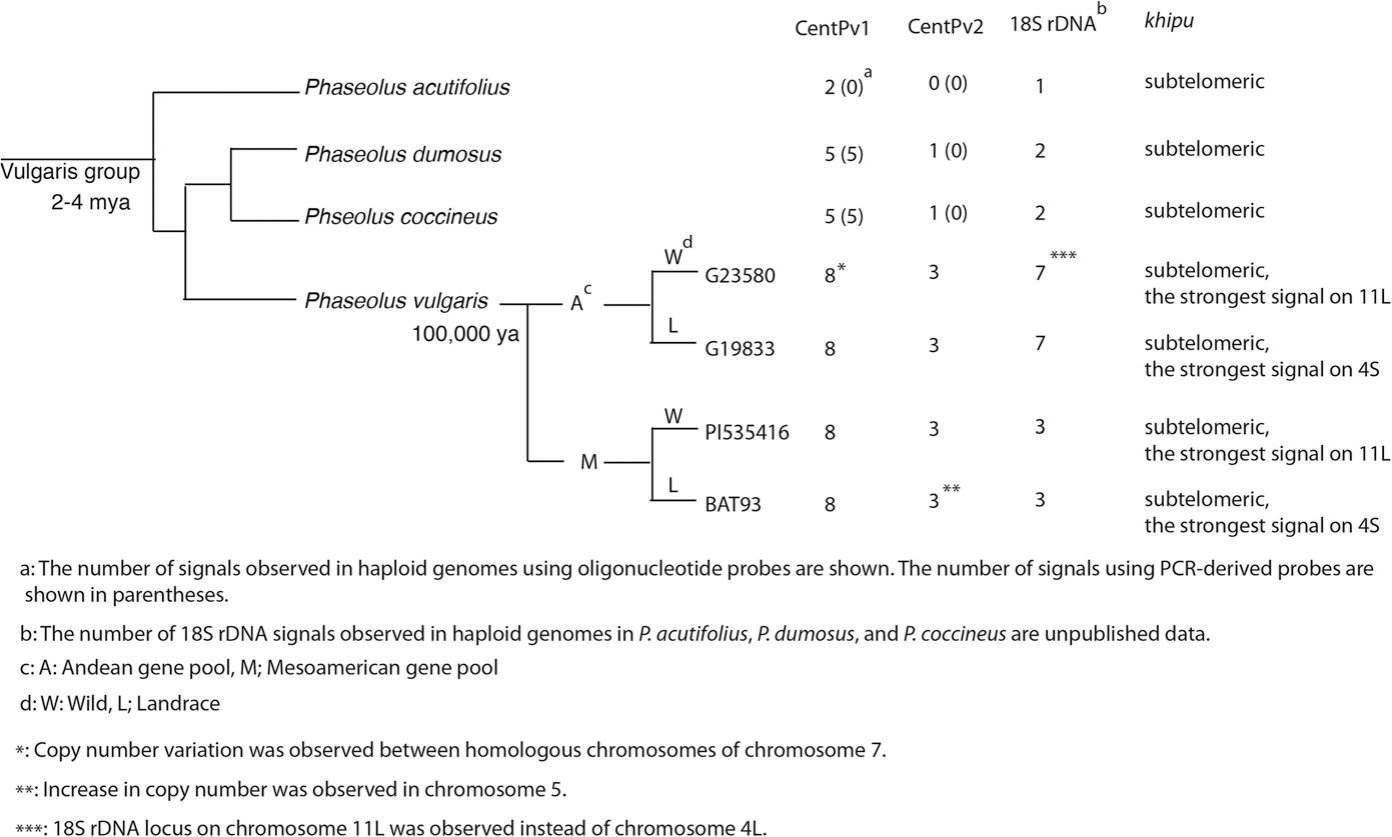
Summary of FISH-based karyotyping in common bean and *Phaseolus* species.

In summary, FISH-based karyotyping provides insights into the stability of chromosomal structures and the evolution of satellite repeats, both copy number and distribution, as shown for centromeres and subtelomeres within common bean, and variability among closely related *Phaseolus* species (Figure 7). Our data indicates that, at the cytological level, satellite repeats are evolving actively and rapidly and contribute to the unique chromosomal structures of individual species. Although we observed changes in these structures, the group as a whole appeared to be much more “stable” than their maize counterparts (Kato *et al.* 2004; Albert *et al.* 2010). This stability may be due to differences in breeding systems (outcrossing *vs.* selfing), and the content and activity of transposons. This karyotyping system can be used to trace chromosomal behavior during cell division, detect specific chromosomes involved in trisomics, transgenes, and structural changes such as inversion and translocation, track chromosomes in interspecific crosses, and facilitate breeding strategies that require chromosome identification (Findley *et al.* 2010; Xiong and Pires 2011).

## 

## Supplementary Material

Supplemental Material
