# Supplementary material for: Fluorescence In Situ Hybridization (FISH)-Based Karyotyping Reveals Rapid Evolution of Centromeric and Subtelomeric Repeats in Common Bean (Phaseolus vulgaris) and Relatives
Source: G3 (Bethesda). 2016 Feb 9;6(4):1013–22. doi: 10.1534/g3.115.024984 (PMC4825637; doi:10.1534/g3.115.024984)
Supplement: Supplemental Material [file supp_6_4_1013__index.html]

Fluorescence In Situ Hybridization (FISH)-Based Karyotyping Reveals Rapid Evolution of Centromeric and Subtelomeric Repeats in Common Bean (Phaseolus vulgaris) and Relatives — Supplemental Material 

# Fluorescence *In Situ* Hybridization (FISH)-Based Karyotyping Reveals Rapid Evolution of Centromeric and Subtelomeric Repeats in Common Bean (*Phaseolus vulgaris*) and Relatives

## Supplemental Material for Iwata-Otsubo *et al.*, 2016

**Files in this Data Supplement:**

- Figure S1 - FISH analysis on G19833 chromosomes. (A) Mitotic metaphase chromosomes counter-stained with DAPI. Bar shows 5 µm. (B) FISH image with a probe mixture of Cy5-CentPv1 (blue), FAM-CentPv1\_A (green), TEX615-CentPv2 (red) and FAM-CentPv2\_A (green). Overlapping signals of Cy5-CentPv1 (blue) and FAM-CentPv1\_A (green) are displayed as dark green on chromosome 8. Overlapping signals of TEX615-CentPv2 (red) and FAM-CentPv2\_A (green) are displayed as yellow on chromosome 11. (.tif, 1,937 KB)
- Figure S2 - FISH analysis of 25-bp oligonucleotide probe targeted to *khipu* on BAT93 chromosomes. (A) Mitotic metaphase chromosomes counter-stained with DAPI. (B) Signals of *khipu*. Allows show the strongest signals on chromosome 4S. Arrowheads show the second strongest signals on chromosome 11L. (C) Merged image. Scale bar indicates 5 µm. (.tif, 1,143 KB)
- Figure S3 - Multiple alignments of CentPv1 and its variant, CentPv1\_C (A) and CentPv1\_D (C). The 25—bp oligonucleotide CentPv1\_B for FISH was designed from the region in the yellow rectangle targeting to the polymorphisms shown by asterisks. (B) FISH analysis of CentPv1 variant, CentPv1\_C (red) and CentPv1 (blue). CentPv1\_C signals overlapped with all CentPv1 signals. Scale bar indicates 5 ?m. (.tif, 6,778 KB)
- Figure S4 - FISH-based karyotyping and the second FISH reprobed with 18S rDNA and BAC clones in accessions G23580 (A and B) and PI535416 (C and D). Scale bars indicate 5 µm. (A and C) FISH-based karyotyping. These chromosomes are aligned and shown in Figure 4. (B) FISH using 18S rDNA (green) and chromosome 1 specific BAC clone, 0043E17 (Red). (C) FISH using 18S rDNA (green), chromosome 4 specific BAC clone, 0095D15 (green), and chromosome 1 specific BAC clone, 0043E17 (Red). Chromosomes 4 recognized by 0095D15 signals are shown with arrowheads. (.tif, 5,482 KB)
- Figure S5 - FISH images of PCR amplified CentPv1 signals (green) on mitotic chromosomes (blue) of *P. coccineus* (A and B) and *P. dumosus* (C and D). Bar represents 5 µm. (.tif, 3,184 KB)
