# Supplementary figures and images for: Fluorescence In Situ Hybridization (FISH)-Based Karyotyping Reveals Rapid Evolution of Centromeric and Subtelomeric Repeats in Common Bean (Phaseolus vulgaris) and Relatives
Source: G3 (Bethesda). 2016 Feb 9;6(4):1013–22. doi: 10.1534/g3.115.024984 (PMC4825637; doi:10.1534/g3.115.024984)

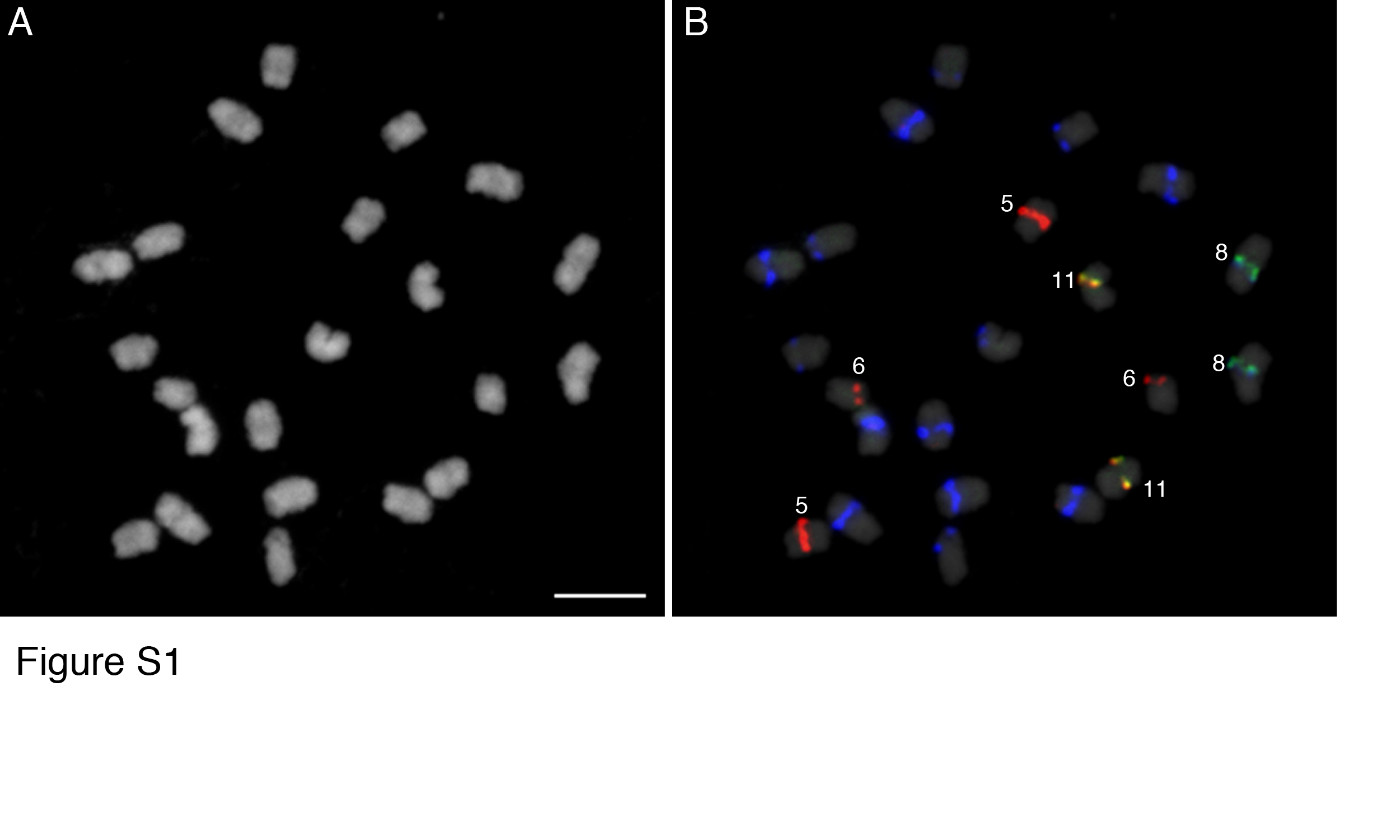

Supplement: Supplemental Material [file supp_g3.115.024984_FigureS1.tif]

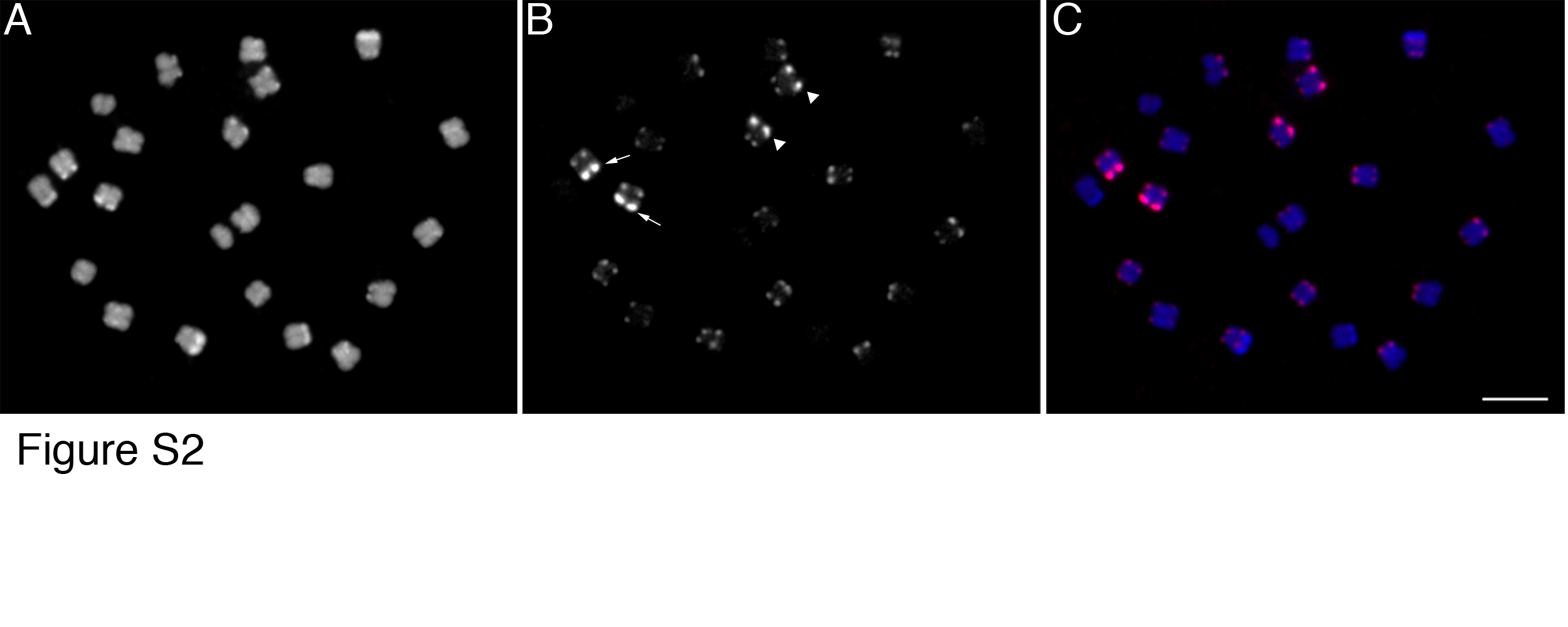

Supplement: Supplemental Material [file supp_g3.115.024984_FigureS2.tif]

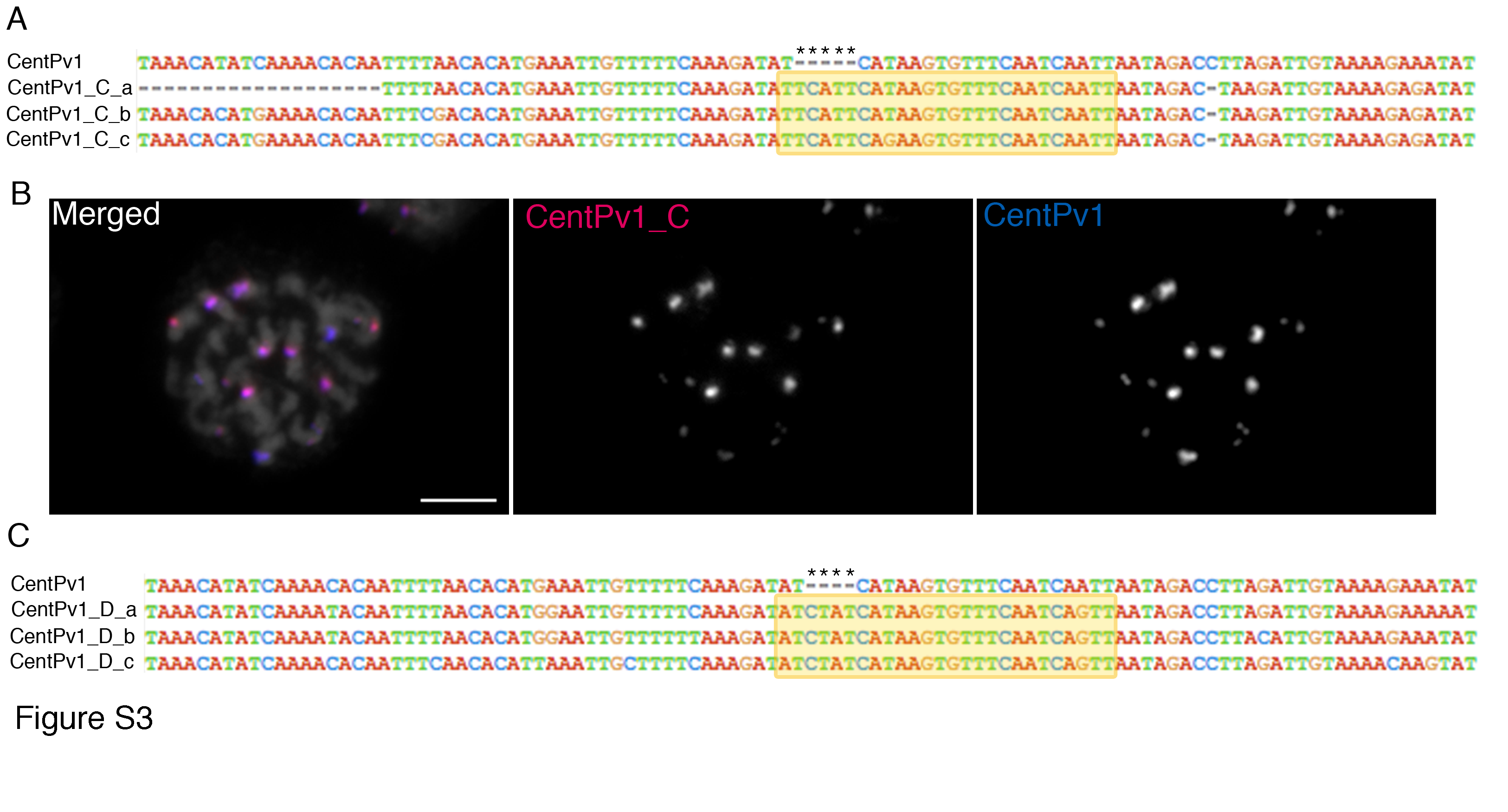

Supplement: Supplemental Material [file supp_g3.115.024984_FigureS3.tif]

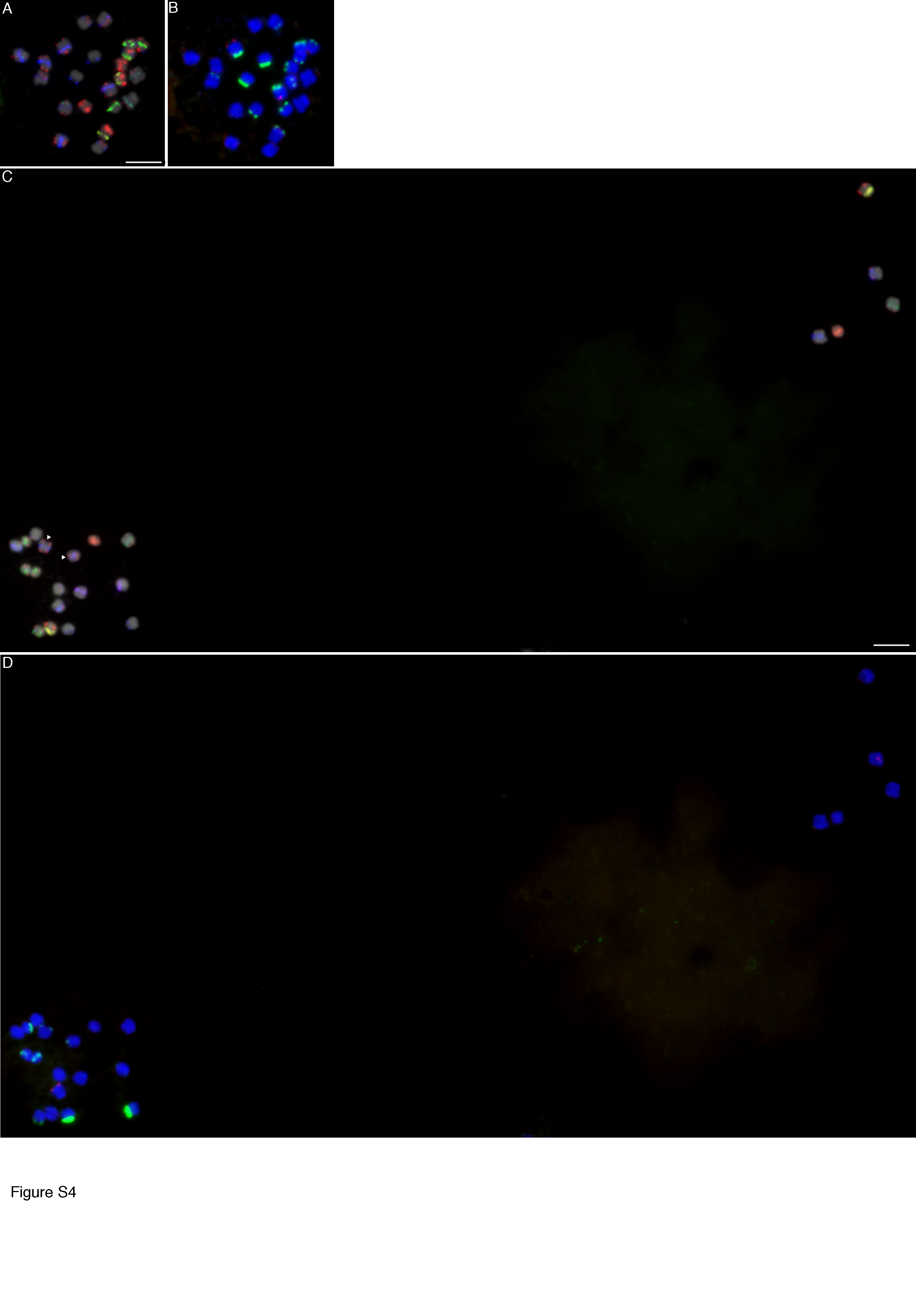

Supplement: Supplemental Material [file supp_g3.115.024984_FigureS4.tif]

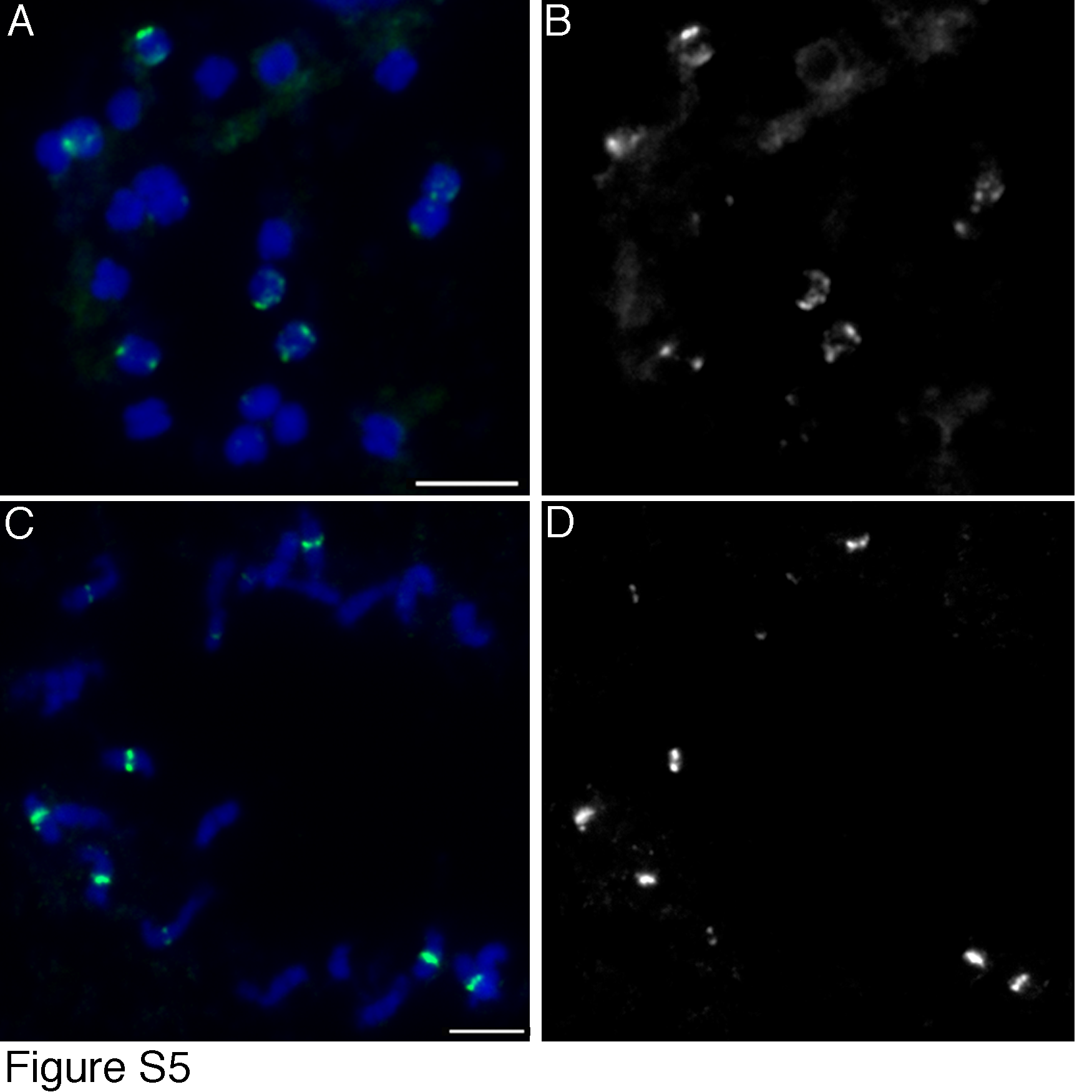

Supplement: Supplemental Material [file supp_g3.115.024984_FigureS5.tif]
